# Effect of Soundscape Augmentation on Behavioral Symptoms in People With Dementia: A Pilot Randomized Controlled Trial

**DOI:** 10.1093/geroni/igae069

**Published:** 2024-08-05

**Authors:** Arezoo Talebzadeh, Dick Botteldooren, Pieter Thomas, Steven Stewart, Dominique Van de Velde, Patricia De Vriendt, Paul Devos, Andrea Iaboni

**Affiliations:** Department of Information Technology, WAVES—Ghent University, Ghent, Belgium; Department of Information Technology, WAVES—Ghent University, Ghent, Belgium; Department of Information Technology, WAVES—Ghent University, Ghent, Belgium; KITE—Toronto Rehabilitation Institute, University Health Network, Toronto, Ontario, Canada; Faculty of Mediciness and Health Care Sciences, Department of Rehabilitation Sciences, Occupational Therapy Research Group, Ghent University, Ghent, Belgium; Faculty of Mediciness and Health Care Sciences, Department of Rehabilitation Sciences, Occupational Therapy Research Group, Ghent University, Ghent, Belgium; Occupational Therapy Department, Artevelde University of Applied Sciences, Ghent, Belgium; Department of Information Technology, WAVES—Ghent University, Ghent, Belgium; KITE—Toronto Rehabilitation Institute, University Health Network, Toronto, Ontario, Canada; Temerty Faculty of Medicine, Department of Psychiatry, University of Toronto, Toronto, Ontario, Canada

**Keywords:** Neuropsychiatric symptoms, sound environment, Nonphamacological interventions

## Abstract

**Background and Objectives:**

Sound is an important environmental factor that influences the expression of behavioral and psychological symptoms of dementia. Recent research on the effect of soundscape has shown promising results in improving environmental impact on people with dementia. However, no controlled studies have aimed to quantify the effects of soundscape intervention on resident outcomes. The aim of this study was to assess the feasibility and impact of a soundscape intervention on people with dementia and behavioral symptoms.

**Research Design and Methods:**

Pilot single-blind repeated-measures randomized controlled trial of an augmented soundscape intervention. Participants were people with dementia in a hospital-based specialized dementia unit. Participants were randomized to an augmented soundscape intervention delivered in their room in the morning and evening or treatment as usual, with 2 baseline weeks and 4 weekly post-randomization assessments of the primary and secondary behavioral outcomes.

**Results:**

The soundscape intervention was feasible in terms of recruitment, retention, and delivery of the intervention. There were improvements in the neuropsychiatric inventory total scores over time in both groups (−5.89, 95%CI −8.45 to −3.28, *p* < .001), but no differences between groups. There were no significant group, time, or group × time differences for the Pittsburgh Agitation Scale (PAS) total score. For the PAS-resisting care subscale, there was a significant group × time difference, with a greater reduction in the soundscape group over the study period (−0.81, 95% CI −1.59 to −0.03, *p* = .042).

**Discussion and Implications:**

In this pilot study, soundscape augmentation was a feasible and effective nonpharmacological approach to reducing resistance to care in people with dementia, although it did not improve neuropsychiatric symptoms more globally. Further studies with larger samples and of longer duration are needed to investigate the long-term effects of augmented sonic environments on people with dementia.

**Clinical Trials Registration Number:**

NCT04809545

**Translational Significance:** This pilot study used soundscape augmentation as a non-pharmacological approach to reduce behavioural and psychological symptoms of dementia in a care facility for people with dementia. Implementing sound augmentation through a series of sound segments played at a specific time improved resistance to care. The study confirms the acceptability of a soundscape intervention and the positive effect of designed soundscape on people with dementia.

## Background and Objectives

Many factors contribute to the expression of behavioral symptoms of dementia, with an important factor being the design of the dementia care environment (Quirke et al., 2023). In particular, noise is an important environmental factor that influences the expression of behavioral and psychological symptoms of dementia (BPSD), affecting sleep, agitation and anxiety, among other symptoms (Fleming et al., 2016). Sources of noise in dementia care settings include environmental noise such as door slamming, alarms, telephone ringing, and human vocal and nonvocal sounds (Aletta et al., 2018; Janus et al., 2021; Talebzadeh, Decoutere, et al., 2023; Thomas et al., 2020). Studies have found that noise levels can reach 54–67 dB in living, dining and residents’ rooms of nursing homes, and dementia care facilities (Aletta et al., 2017; Brown et al., 2016; Devos et al., 2020; Jerlehag et al., 2018; Peng et al., 2018). Unwanted sounds can be disorienting, trigger distress, and agitation, and have a negative impact on the quality of life of people with dementia (Brown et al., 2016; Van Den Bosch & Andringa, 2014).

In the last few decades, insights into the impact of the sonic environment on persons have expanded to include not only the damaging effect of noise but also the valuable effects of a well-designed sonic environment (Kang & Schulte-Fortkamp, 2010; Schulte-Fortkamp et al., 2023). Sound is an essential sensory stimulus that helps self-orientation in space and time and triggers positive and negative or traumatic memories. “Soundscape”(ISO12913-1, 2014; Schafer, 1977) refers to an acoustic environment perceived and experienced by a person or people within a specific context. A well-designed soundscape can improve the quality of life and create a positive health impact (Schulte-Fortkamp, 2021). Hence, soundscape is considered a beneficial environmental factor in promoting health. The literature shows that natural and nonnatural soundscapes positively affect people with severe intellectual disabilities (Andringa & Van Den Bosch, 2013). A well-designed soundscape promotes safety (Van Den Bosch & Andringa, 2014), influences mood, and triggers specific actions (Devos et al., 2019); for example, a slightly audible sound of turning the page of a book, a breeze through leaves or domesticated animals and birds brings the feeling of safety and reduces the boredom of an acoustic environment (Van Den Bosch & Andringa, 2014). Familiar and recognizable sounds in the soundscape, in particular bird sounds, water streams, and human interaction sounds such as restaurant and café, have been found to have the highest favorable ratings by staff in nursing homes (Talebzadeh, Botteldooren, et al., 2023).

The latest research on the effect of the soundscape has shown some promising results in improving the impact of the environment on people with dementia. Aletta et al. conducted a sound level and soundscape quality monitoring study in nursing homes’ living rooms. They identified the potential role of a sonic environment in promoting quality of life for people with dementia (Aletta et al., 2017). Thomas et al. showed the effect of acoustic intervention on soundscape perception in nursing homes, improving pleasantness and providing a quieter indoor soundscape (Thomas et al., 2020). Other research has used technology to enhance the soundscape of care facilities. Kosters et al. used a mobile application to evaluate the soundscape of nursing homes and improve nursing staff sound awareness; they then implemented “micro-interventions“ to reduce disturbing sounds and found improved staff ratings of soundscape (Kosters et al., 2022). In an observational study, De Pessemier et al. looked at the effect of augmented soundscape on people with dementia in Flanders nursing homes (De Pessemier et al., 2022). They reported a significant increase in positive caregiver feedback for 6 out of the 19 participants exposed to the soundscape. Previous research is limited by small sample sizes and observational designs, and to date, no controlled studies have aimed to quantify the impact of a soundscape intervention on resident outcomes.

This pilot randomized controlled trial aimed to assess the feasibility of a clinical trial of a soundscape intervention in a specialized dementia care environment and determine the effect size of this intervention on outcomes related to BPSD. The intervention focuses on older adults with dementia and BPSD. The soundscape intervention involved playing sounds to augment the existing sonic environment in the patient’s room in the early evening and morning. The feasibility outcomes were the recruitment, retention, and delivery of intervention. The primary outcome was the total neuropsychiatric inventory (NPI), with secondary outcomes of the Pittsburgh agitation scale (total and resisting care subscale) and NPI agitation and aggression subscales.

## Research Design and Methods

### Study Design

We designed a single-blind, repeated-measure pilot randomized clinical trial for feasibility and to estimate the treatment effect over the evaluation period. We used a 2:4:1 design, with baseline measures captured for two weeks. The baseline assessment was followed by randomization to *Soundscape* or *TAU* (treatment as usual), with four weekly outcome measurements and one-week post-intervention assessment. A 2,4,6 block randomization (Efird, 2011) and the sealed envelope randomization application (Sealed Envelope Ltd., 2022) were used to randomize participants. Randomization was conducted by a researcher located off-site who had no involvement in recruitment, intervention delivery, or data collection. The protocol was pre-registered on ClinicalTrials.gov (NCT04809545). The CONSORT (CONsolidated Standards Of Reporting Trials) checklist is available as Supplementary Material (Eldridge et al., 2016).

### Participants and Setting

This study occurred in the Specialized Dementia Unit (SDU) at Toronto Rehab, University Health Network in Toronto, Canada between June 2021 and June 2022. It is a 17-bed psychogeriatric unit which admits people with moderate to severe dementia from long-term care or nursing homes to treat BPSD and has an average length of stay of 60 days. Participant recruitment occurred via a centralized recruitment process whereby all substitute decision-makers of incapable patients are approached at admission to ask about interest in research. Those who indicated interest were provided an opportunity to consent to the research study. The inclusion criteria were 65 years or older, diagnosis with dementia, symptoms of BPSD at baseline and English speaking. The exclusion criteria were severe hearing impairment and those receiving end-of-life treatment. We used the Simple Hearing Test (Pirozzo et al., 2003) to establish if severe hearing impairment was present. The initial protocol had admission to a private room on the unit as an inclusion criterion; however, due to the circumstances of the COVID-19 pandemic, a change was made to the protocol such that the speaker would follow individuals to a double room if their rooms were changed. The project protocol was reviewed by the University Health Network Research Ethics Board and approved under REB# 20-5067 (2021-03-12).

### Apparatus

The soundscape system consisted of an internet-connected sound player and a feedback button and was connected to the web interface. A more detailed description of the system can be found in De Pessemier et al. (2022). The sound player system (one player installed in each room) had a speaker, a small single-board computer (Raspberry Pi), a clock (PRI RTC Clock Real-Time DS3231SN), a USB with SIM Card for internet connection (to avoid Wi-Fi drop-off). The player system was about 200 mm  ×  200 mm  × 120 mm in size, was placed on top of a shelf or a bedside table close to the bed, and plugged into an outlet (see Supplementary Figure 1 for a photograph of the set-up). Each device has a unique device ID.

The web interface gave access to the overall soundscape control and allowed the initial composition of the soundscape and the daily schedule of all rooms’ different player systems. The interface was connected to a cloud-based server and provided site-level control of the system, allowing the activation and deactivation of the players remotely. The server was connected to all the rooms’ sound player systems. Each device was labeled in the server by a device ID, and the room number was identified in the server based on each device. The first author had access to the interface to monitor the players daily and ensure that intervention was being delivered. If a malfunction happened (e.g., the accidental unplugging of a sound player), the researcher would communicate the problem with the research assistant onsite. The research assistants had no access to the interface.

### Soundscape Group

Participants were randomized using block randomization after a two-week baseline period to Soundscape or TAU. This study was single-blind, where the assessor and responsible physician were blinded to the treatment, whereas nursing staff, patients, and family were not blinded, as they were exposed to the soundscape. Nursing staff were instructed to communicate with the assessor or physician about the study group assignment only if a clinically important event related to the soundscape occurred. To prevent inadvertent unblinding, the intervention was delivered between 7 p.m. and 7 a.m., outside the usual physician and research assistant hours on the unit. The timing of the intervention delivery was chosen for a few reasons: residents are not in their rooms during the day and would not be exposed to the intervention. Residents move around more during the day; therefore, there is more risk that the TAU group would be exposed to the intervention. Finally, the timing allowed the physicians and research assistants to be blinded to the intervention. The timing influenced the design of the soundscape based on tasks required during those hours: getting prepared for bed, relaxing and sleeping, waking up and getting ready for the day, and receiving morning care. The sound played from 7:30 pm to around midnight and then from around 4 a.m. to 7 a.m. Sounds used at night (such as rainfall and crickets) were chosen for their potential to mask the unwanted noise of the care unit at night, consistent with the wider use of white noise for sleep (Riedy et al., 2021).

### Control Group

As part of usual care, patients in the SDU receive a comprehensive assessment of their health and symptoms of dementia involving consultation by a geriatric psychiatrist, hospitalist, physical therapist, occupational therapist, and recreation therapist, and pharmacological and nonpharmacological treatment plans. All participants in the study received this standard of care.

### Study Outline

**Figure 1.**
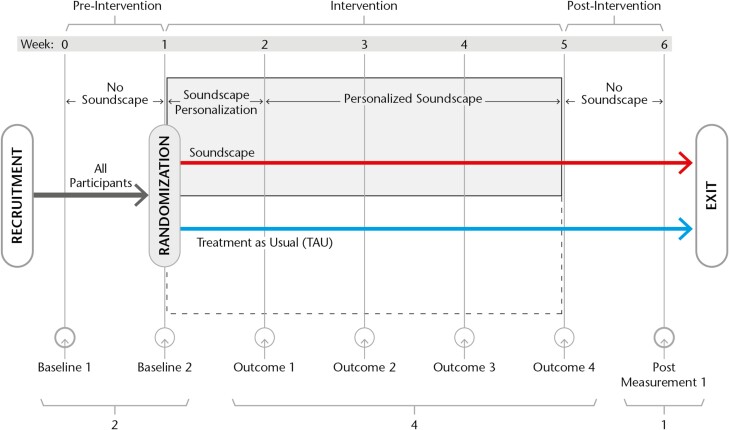
Study outline: six weeks from recruitment to exit, with two baseline measurements, four weekly outcome measurements, and one post-intervention measurement.

All participants underwent a baseline assessment at study entry and had a one-week evaluation before randomization. After randomization to soundscape intervention, they had one week of adjustment to the soundscape and three weeks of exposure to the soundscape. At the beginning of week six, the soundscape system was turned off, and an exit assessment took place one week later (Figure 1). Outcome measures were assessed weekly: two pre-interventions, four interventions, and one postintervention (2 4:1).

### Baseline Measure

All participants were characterized at baseline 1 using demographic measures (age, sex) and several study-specific assessments: Mini-Mental State Examination (Folstein et al., 1975), Severe Impairment Rating Scale (Rabins et al., 1996), Performance Oriented Mobility Assessment (Tinetti, 1986), Quick Dementia Rating System (Galvin, 2015), Clinical Frailty Scale (Rockwood et al., 2005).

### Primary Outcome

The primary outcome was the total Neuropsychiatric Inventory Questionnaire-Clinician (NPI-C; Kaufer et al., 2000), recorded by the research assistant weekly after reviewing the totality of weekly behavior by the participant, including a review of the written chart and discussion with clinical staff.

### Secondary Outcomes

Secondary outcomes were the Pittsburgh Agitation Scale (PAS) total score and the resistance to care subscale (Rosen et al., 1994). The PAS is a long-standing standard-of-care assessment on this unit and is completed by nurses and entered into the nursing flowsheet once per shift (day, evening, and night). It documented the severity of four different behaviors (resisting care, aggression, motor agitation, and aberrant vocalization) on a scale from 0 to 4, with scores of 3 or 4 representing clinically important behaviors. The total number of scores of 3 or 4 given in a week for all behaviors and for the “resistance to care” item were captured as secondary outcomes. Although the evening and night nurses were not blinded to the intervention and could hear the sounds, they were unaware that information from their flowsheet was being captured as part of the study outcome.

### Procedure

As part of a previous ethnographic study (Talebzadeh, Decoutere, et al., 2023) and co-design sessions with nursing home residents, their caregivers, and staff in multiple nursing homes (Devos et al., 2018), the research team understood the preferred sounds that positively affect residents with dementia. The team previously collected and assessed a set of sounds to design the soundscape (Talebzadeh, Botteldooren, et al., 2023). This collection included natural sounds, anthropogenic sounds (from human activities), and music. The sound segments with saliency or sharpness were used for activation in the morning (bird chorus, e.g.), whereas sounds with masking characteristics, such as crickets and streams, were used during sleep to mask the unwanted noise of the corridors and other activities in the unit. For each typical activity in a dementia care environment, a collection of sound segments was selected to be played. In a previous study by research and based on the earlier co-design session, 17 activities were selected as typical day activities in a dementia care setting. For example, waking up, washing and dressing, having breakfast, showering, and getting ready for sleep. The research team then categorizes each sound segment in relation to these activities (Talebzadeh et al. 2023.) The system randomly chose a segment from the list of sounds assigned to a specific activity/time of day. All sound segments were played at a level close to the expected background (about 46 dBA), so they attract attention but won’t be perceived as louder than the background. The activities investigated in this study included preparing for bedtime, resting or sleeping, falling asleep, waking up, washing, and dressing.

Additionally, the system featured a button that allowed participants, staff, or caregivers to silence specific sounds if they found them unpleasant or disruptive. The use of this button would also help personalize the soundscape by excluding muted sounds. Each participant had a sound player system installed in their private rooms. The player would play the set of sounds for those randomized to *Soundscape* intervention, and no sound would play for those in *TAU*. No significant system disruption occurred during this study. The first author would monitor the interface to ensure that the player was plugged in and that the soundscape played during the intervention. The research team had a backup system to use in case of malfunction.

### Analysis

The baseline demographics of study participants were summarized descriptively. We used generalized linear mixed-effects models (intention-to-treat) to analyze the primary and secondary outcomes. This model allowed an analysis that accounted for repeated measures of longitudinal data with heterogeneity or variability among subject-specific response profiles and missing data. For this analysis, we used data from the baseline 2 timepoint as the baseline and the 4 postrandomization outcome timepoints.

## Results

### Feasibility

**Figure 2.**
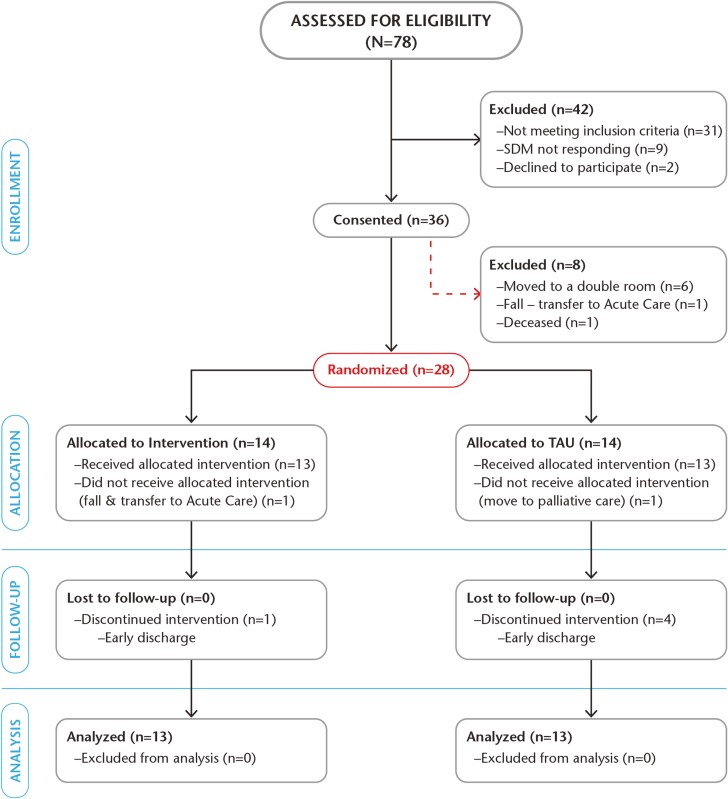
Consort diagram showing study recruitment; randomization into the intervention group and TAU (treatment as usual) group. SDM = substitute decision-maker; TAU = Treatment As Usual.

The CONSORT diagram (Figure 2) shows the study recruitment. In the end, 28 individuals were randomized. Two participants dropped out after randomization without receiving the intervention, leaving 13 individuals to be analyzed per group. Five participants in the intervention group and six in the TAU group did not complete the study (all dropped out due to early discharge from the unit). All participants were included in our intention-to-treat analysis.

### Participants

Table 1 shows the characteristics of the study participants. The randomization was effective, with no significant differences between the two groups, although there were slightly more men and younger patients in the Soundscape group. The participants had moderate-severe BPSD at baseline (NPI total mean 34.8 ± 4.7) and severe cognitive impairment (MMSE mean 6.5 ± 1.3).

**Table 1.** Participant Baseline Characteristics.

Characteristics	Soundscape *n *= 13			TAU *n* = 13			chi2/T	df	*p*
	*n* (%)	Mean	SD	*n* (%)	Mean	SD			
Sex (Women)	4 (31%)			7 (54%)			1.14		.23
Age		76.9	2.4		82.8	2.0	−1.9	24	.072
MMSE		5.6	8.0		7.3	4.9	−0.6	21	.562
SIRS		13.5	9.1		18.6	6.1	−1.6	21	.130
QDRS		22.5	8.1		20.0	10.2	0.7	24	.502
CFS		6.8	1.1		7.0	0.4	−0.5	22	.683
NPI-C (total)		33.3	22.6		36.2	25.6	−0.3	24	.760
Agitation		6.2	4.8		5.8	5.3	0.2	24	.878
Aggression		2.5	3.6		2.6	3.9	−0.1	24	.918
PAS (total)		28.2	19.3		29.8	21.6	−0.2	24	.849
Resisting Care		8.0	6.9		6.8	7.7	0.4	24	.690

*Notes*: TAU = treatment as usual. MMSE: Mini-mental State Examination; maximum score of 30 (Folstein et al., 1975). SIRS: Severe Impairment Rating Scale consists of 14 items representing three cognitive areas: memory, language, and motor, with a maximum score of 28 (Rabins et al., 1996; Yeo et al., 2016). QDRS: Quick Dementia Rating System is a 10-item questionnaire with scores ranging from 0 to 30, with higher scores representing more significant cognitive impairment (Galvin, 2015)^.^ CFS: Clinical Frailty Scale assesses frailty, scoring from 1 (very fit) to 9 (terminally ill; Rockwood et al., 2005). NPI-C: Neuropsychiatric Inventory Questionnaire-Clinician (De Medeiros et al., 2010). PAS: The Pittsburgh Agitation Scale; count of number of scores of 3 or 4 (clinically important agitation) over 21 shifts rated during the week (Rosen et al., 1994).

### Outcomes

**Figure 3.**
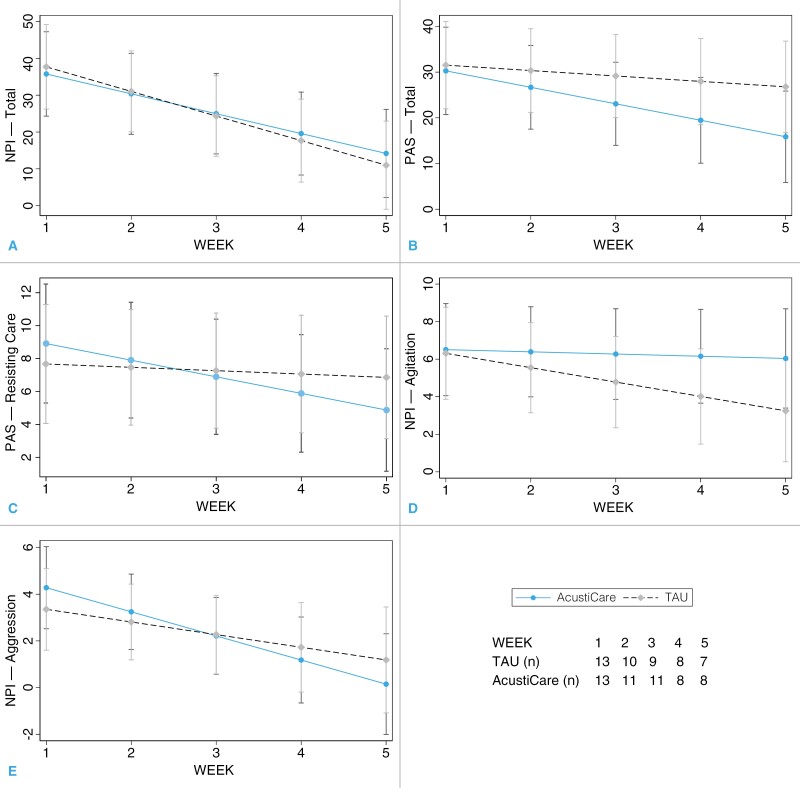
Adjusted prediction of group with 95% CIs for (**a**) NPI-total, (**b**) PAS-total, (**c**) PAS-resisting care, (**d**) NPI-agitation, and (**e**) NPI-aggression. NPI = Neuropsychiatric Inventory Questionnaire-Clinician; PAS = Pittsburgh Agitation Scale; TAU = Treatment As Usual.

In a linear mixed effects model (Table 2), there were improvements in the NPI-C total scores over time in both groups (−5.89, 95% CI –8.45 to −3.28, *p* < .001), but no group or group × time differences (Figure 3). For the PAS total score, there were no significant group, time, or group × time interactions, with a trend to reduced PAS total score in the *Soundscape* group (−2.42, 95% CI –4.97 to.13, *p* = .06).

**Table 2.** Results of Linear Mixed Effect Models for Primary and Secondary Outcomes, Comparing TAU and Intervention Groups.

Variable	Coeff	95% CI		Z	p
NPI-C total					
Time	−5.89	−8.49	−3.28	−4.43	<.001
Group	−5.18	−22.40	12.04	−0.59	.555
Group × time	1.78	−1.81	5.37	0.97	.332
PAS total					
Time	−1.19	−2.99	0.62	−1.29	.197
Group	1.17	−13.35	15.70	0.16	.875
Group × time	−2.42	−4.97	0.13	−1.86	.063
PAS resisting care					
Time	−0.20	−0.75	0.35	−0.73	.467
Group	2.05	−3.31	7.41	0.75	.454
Group × time	−0.81	−1.59	−0.03	−2.03	.042
NPI Agitation					
Time	−0.76	−1.20	−0.33	−3.44	.001
Group	−0.45	−4.09	3.18	−0.24	.807
Group × time	0.65	0.05	1.25	2.12	.034
NPI Aggression					
Time	−0.54	−1.11	0.02	−1.89	.059
Group	1.42	−1.46	4.29	0.97	.334
Group × time	−0.49	−1.27	0.29	−1.23	.217

*Notes*: NPI-C total: Neuropsychiatric Inventory Questionnaire-Clinician total score (De Medeiros et al., 2010). PAS-Total: The Pittsburgh Agitation Scale (Rosen et al., 1994), the total number of ratings of 3 or 4 in the week. PAS Resisting Care: Total ratings of 3 or 4 for resisting care in a week. NPI-C Agitation: agitation subscale of NPI-C. NPI-C Aggression: aggression subscale of NPI-C.

For the PAS-resisting care subscale, there were no group or time differences but a significant group × time difference, with a greater reduction in PAS-resisting care score in the *Soundscape* group (−0.81, 95% CI −1.59 to −.03, *p* = .042). There was an improvement over time in both groups in the NPI-Agitation subscale but a greater improvement over time in the *TAU* group compared with soundscape (0.65, 95% CI 0.05 to 1.25, *p* = .034). There was a nonsignificant improvement over time in both groups in the NPI-Aggression, with greater improvement in the *Soundscape* group (−0.49, 95% CI −1.27 to 0.29, *p* = .22).

### Effect size

Based on the findings, the group-by-time interaction uniquely accounted for 1% of the variance of the NPI-C total score and 3% of the variance in PAS-resisting care as measured by Cohen’s *f*, which is a small effect size. Using Stata 16 to calculate a required sample size for an RCT based on the means of each group over time, correlations of repeated measures, and the error variance observed in this pilot study for a study using the PAS-resisting care as a primary outcome and two groups, five repeated measures, alpha error probability = 0.05, and 0.8 power, a sample of 38 would be needed, and with 0.9 power, a sample of 48 would be required.

## Discussion

This study is the first pilot randomized controlled trial of a soundscape intervention for older adults with dementia with the aim of improving BPSD. Recruitment was feasible, and there were no adverse effects or outcomes related to the intervention. We demonstrated that the intervention was safe and highly acceptable, with a high recruitment rate. Overall, there was no difference between groups on the NPI-C total score primary outcome, but there was an improvement in the secondary outcome of PAS-resisting care, with a small effect size. Previous research showed the positive effect of soundscape intervention in nursing homes by improving BPSD (De Pessemier et al., 2022; Devos et al., 2018; Talebzadeh et al., 2022), which aligns with our findings.

There may be several explanations for the differences between the primary outcome (NPI-C, research assistant assessed) and secondary outcome (PAS, taken from nursing flowsheets). One possibility is that nurses who worked on the evening and night shifts were unblinded to the study because they could hear the sounds playing in participant’s rooms. This exposure may have biased their ratings of behaviors unconsciously. However, it is important to note that the nursing staff were unaware that the PAS charting was an outcome measure in the study. It is also important to note the differences between what is measured by the PAS and NPI scales, where PAS is a count of the number of care shifts in a week where clinically important symptoms are exhibited.

In contrast, NPI provides a score based on the product of the weekly frequency and severity of the behavior. We had initially selected the NPI-C as the primary outcome measure, as we hypothesized that their experience of the evening and morning soundscape would, directly and indirectly, affect their behavioral symptoms throughout the week. However, the PAS was included as a secondary outcome, as we also hypothesized that the soundscape would have a more immediate and direct impact on behaviors during the provision of care, which would take place during the delivery of the evening and morning intervention. The pilot study has helped to identify that a more direct measure of the behaviors during the care episode may be a better outcome measure for this type of intervention compared with a neuropsychiatric symptom scale.

Several unanticipated challenges arose due to the COVID-19 pandemic. The study was designed to recruit patients assigned to the 12 private rooms on the unit; during the pandemic, these rooms were required to isolate symptomatic or infected individuals, and thus, room changes were frequent, which required a change in the protocol such that one occupant of a double room could be included in the study. Before this change to the inclusion criteria was made, 20 individuals were excluded as they were not in a single room. Six more individuals were excluded after providing consent due to a move to a double room. Overall, we achieved a 76% recruitment rate of eligible patients.

The pandemic added further challenges regarding intermittent outbreaks on the unit and periodic isolation of symptomatic residents when we could not conduct study assessments, resulting in some missing data. However, this was mitigated by the repeated measures design. No adverse events were recorded during the study, no participants withdrew, and the intervention and study were highly acceptable to families, patients, and staff. No issues related to soundscape disturbing a roommate were reported. We did not identify any incidents of unblinding of the research assistant.

A strength of this study was that it examined a focused and augmented soundscape intervention that was delivered at times when the patient was most likely in their room and receiving care. In the morning, sounds with clear and dynamic characters (Talebzadeh, Botteldooren, et al., 2023), such as bird sounds, were chosen to activate the mind. Sounds with high-frequency components trigger abruptness and mood change, resulting in a positive reaction (Hales Swift & Gee, 2017). The sharpness of sound also makes it recognizable from the sonic background and brings attention to the sound; sharpness is also an indicator of the “pleasantness” of a sound (Hales Swift & Gee, 2017). White noise-like sounds, such as cricket or gentle rainfall, were played to prepare the participants for rest and sleep. These sounds can either be perceived as tranquil or masking to cover the disturbing noise of the unit outside the participant’s rooms (Talebzadeh, Botteldooren, et al., 2023).

Environmental intervention in dementia care has been studied, and the positive effect of some of the interventions on agitation and BPSD is well-known (Oliveira et al., 2015). For example, bright light therapy (Burns et al., 2009; Tan et al., 2022) and aromatherapy (Agatonovic-Kustrin et al., 2020; Burns et al., 2011; Yang et al., 2015) positively affect agitation. Music therapy has also been part of dementia care practice to improve mood and lower BPSD, with primarily short-term improvements (McDermott et al., 2013, 2014). Soundscape augmentation as a subtle sonic background can be beneficial not only for people with dementia but also for their caregivers by reducing BPSD and should be part of the design for dementia care.

In terms of limitations, due to pandemic-related factors, we were unable to recruit our total planned sample. However, the final sample size was appropriate for a pilot study and has provided information to help support and plan a full-scale RCT. Outcomes should be more closely tied to the intervention, such as evaluating the effect of the soundscape on the patient’s mood and behaviors during care provision when the individual is exposed to the soundscape. We did not gather stakeholder feedback as part of this study, although the acceptability of this soundscape intervention has been explored in previous observational studies (De Pessemier et al., 2022). Future studies can also use location tracking systems within care homes to quantify better the exposure or “dose” of soundscape and how individuals behave in relation to soundscape (approaching or avoiding the sounds; Haslam-Larmer et al., 2022).

## Conclusion

In this pilot study, soundscape augmentation was a feasible and effective nonpharmacological approach to reducing resistance to care in people with dementia. As soundscape study in healthcare is new, there is a need for methodology improvement. Longitudinal studies are necessary to investigate the long-term effect of an augmented sonic environment on people with dementia. Also, future studies with larger samples are recommended to confirm our findings and extend the knowledge about the effectiveness of soundscape on people with dementia.

## Supplementary Material

igae069_suppl_Supplementary_Material

## Data Availability

The dataset used in this study is not publicly available as the participants did not consent to publish their data. Aggregate, de-identified data are, however, available from the authors upon reasonable request.
